# Exploring the association between dietary fiber intake and hepatic steatosis: insights from NHANES

**DOI:** 10.1186/s12876-024-03256-1

**Published:** 2024-05-10

**Authors:** Xingxing Chen, Liying Fu, Zhongxin Zhu, Yunchao Wang

**Affiliations:** 1 Clinical Research Center, Xiaoshan Affiliated Hospital of Wenzhou Medical University, Hangzhou, Zhejiang 311200 P.R. China; 2 Voluntary Blood Donation Service Center of Xiaoshan District, Hangzhou, Zhejiang 311200 P.R. China; 3 Department of General Practice, The First People’s Hospital of Xiaoshan District, No. 199 South Shixin Road, Hangzhou, Zhejiang 311200 P.R. China

**Keywords:** Controlled attenuation parameter, Hepatic steatosis, Dietary fiber intake, NHANES, Cross-sectional study

## Abstract

**Purpose:**

The link between dietary fiber intake and Non-alcoholic fatty liver disease (NAFLD) is under exploration, yielding inconsistent findings. Considering the limitations of previous research and the significance of dietary fiber in hepatic steatosis, this study investigates the association between dietary fiber intake and Controlled Attenuation Parameter (CAP) among 5935 participants from the National Health and Nutrition Examination Survey (NHANES).

**Materials and methods:**

Multivariable regression was used to evaluate the association between dietary fiber intake and CAP. Smoothed curve fitting and threshold effect analysis techniques were applied to illustrate non-linear relationships.

**Results:**

After adjusting for other variables, a negative correlation emerged between dietary fiber intake and CAP. Subgroup analysis by gender and race/ethnicity revealed a sustained negative association between dietary fiber intake and CAP among females and Whites. Additionally, an inverted U-shaped relationship was observed between dietary fiber intake and CAP among women and other race, with inflection points at 13.80 g/day and 33.45 g/day, respectively.

**Conclusion:**

Our research indicates that in the majority of Americans, there is an inverse relationship between dietary fiber intake and hepatic steatosis. This relationship exhibits an inverted U-shaped curve in women and other race, with a threshold effect. The findings of this study hold potential significance for clinical nutrition interventions, personalized dietary guidance, and advancing research into the diet-disease mechanism relationship.

## Introduction

The most common chronic liver disease worldwide and a major contributor to severe liver disorders is non-alcoholic fatty liver disease (NAFLD) [1]. NAFLD is a multifactorial condition influenced by environmental, genetic, and dietary factors, metabolic imbalances such as insulin resistance, lipid toxicity, inflammation triggered by micro-inflammation, and immune changes, cytokine imbalances, innate immune activation, and alterations in the microbiota [2, 3]. Studies indicate that NAFLD impacts approximately 25% of people globally, significantly burdening healthcare systems [4]. Hepatic steatosis, or liver fat accumulation, is a defining characteristic and primary histological feature of NAFLD [5]. This condition is not only NAFLD’s initial abnormality but also crucial across the disease spectrum [6]. It ranges from simple fat buildup to complex changes such as inflammation, fibrosis, cirrhosis, and liver cancer [7]. Thus, early diagnosis and prevention of hepatic steatosis are essential [8].

Liver biopsy, the gold standard for assessing liver fat, is invasive [9]. To reduce the need for liver biopsy, various non-invasive biomarkers have been studied. While non-invasive biomarkers cannot currently replace liver biopsy and histological assessment, they may play a crucial role in screening patients for liver biopsy [10]. Non-invasive methods such as magnetic resonance imaging (MRI) or computed tomography (CT) scans can assess liver fat [11, 12]. However, they are time-consuming, costly, and not suited for large-scale studies. Transient elastography, favored for NAFLD screening for its precision and non-invasiveness, evaluates hepatic steatosis via the Controlled Attenuation Parameter (CAP) [13, 14]. Numerous studies demonstrate a strong correlation between CAP values and liver fat levels as determined by biopsies [11, 15, 16].

Recently, the influence of diet on health has garnered more attention. Research indicates that consuming more dietary fiber, mainly from grains, fruits, and vegetables, correlates with lower risks of hypertension, hyperuricemia, type 2 diabetes, cardiovascular diseases, and cancer [17–22]. The link between dietary fiber consumption and NAFLD is being explored, with inconsistent results. Some studies indicate lower dietary fiber consumption in NAFLD patients [23–25], whereas others note no significant difference compared to non-NAFLD individuals [26–28]. Variations in study design and populations may explain these discrepancies. Often, these studies diagnose NAFLD using abdominal ultrasonography or the Fatty Liver Index, derived from body mass index (BMI), waist circumference, triglycerides, and gamma-glutamyl transferase. However, these methods do not directly and objectively measure liver fat changes.

The National Health and Nutrition Examination Survey (NHANES) is a program designed to assess the health and nutritional status of American adults and children [29]. It merges interviews and physical examinations, compiling a comprehensive dataset of dietary intake, laboratory data, and health outcomes [30]. The 2017–2020 NHANES introduced transient elastography for the first time to measure liver fat changes, offering the largest U.S. sample of CAP observations. Acknowledging previous studies’ limitations and dietary fiber’s critical role in liver health, this study leverages the broad population from the 2017–2020 NHANES. It employs CAP to explore further the relationship between dietary fiber intake and liver fat changes.

## Materials and methods

### Statement of ethics

All participants gave their informed agreement, and the project was authorized by the National Center for Health Statistics Research Ethics Review Board.

### Study population

**Fig. 1:**
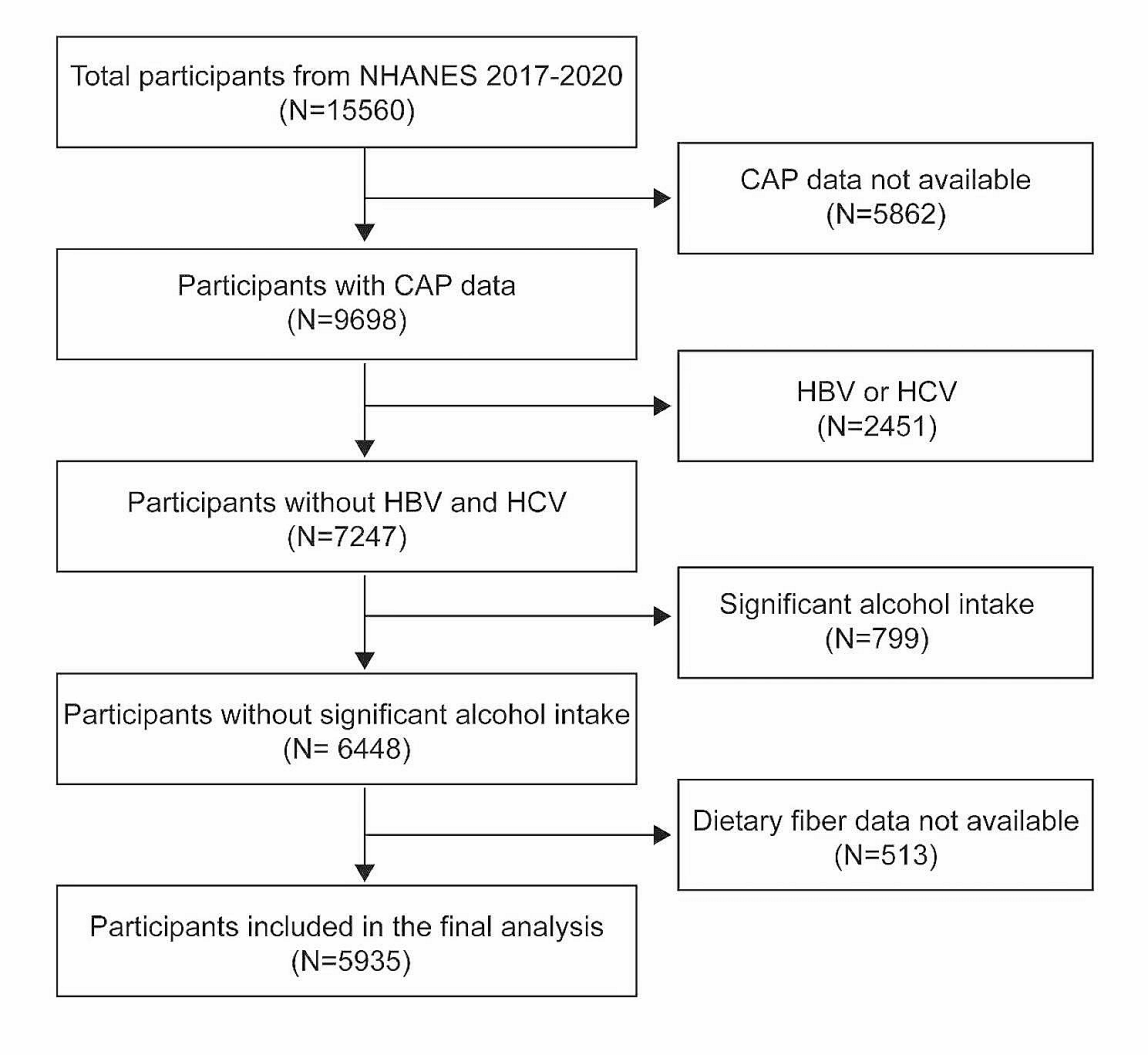
Flowchart of participant selection. NHANES, National Health and Nutrition Examination Survey; CAP, controlled attenuation parameter

In order to achieve nationwide representation, NHANES, a thorough and ongoing cross-sectional national survey in the US, uses a stratified, multistage, clustered random sampling to collect diet and health data from the whole population [29]. Out of 15,560 participants in the 2017–2020 NHANES cycle, 9698 had available CAP data. We excluded 2,451 participants testing positive for hepatitis B antigen, hepatitis C antibody, or hepatitis C RNA, 799 with significant alcohol consumption (4, 5, or more drinks daily), and 513 lacking dietary fiber data. Ultimately, 5935 participants were included in the study. Figure 1 illustrates the sample selection flowchart.

### Variables

The investigation focused on dietary fiber intake as the exposure factor. Dietary fiber intake assessment involved two 24-hour food recall interviews. Three to ten days after the first interview, which took place at a mobile exam facility, there was a telephone interview. The United States Department of Agriculture’s Food and Nutrient Database for Dietary Studies was the source of information on nutrient intakes, including dietary fiber [31]. Dietary fiber intake per participant was averaged from two days of dietary recall data when available, or based on a single day’s data otherwise.

The study’s outcome variable, CAP, was measured using the FibroScan® 502 V2 Touch equipped with liver ultrasonography transient elastography. This device records CAP by measuring ultrasonic attenuation, which reflects hepatic steatosis and indicates liver fatness. According to a recent key study, there is 90% sensitivity in detecting different degrees of hepatic steatosis when CAP values ≥ 274 dB/m, which indicate NAFLD status, are present [32]. This study, which is based on three earlier investigations, classifies CAP ≥ 302 dB/m as a sign of severe steatosis in instances of NAFLD [33–35].

Our study incorporated categorical covariates such as gender, race/ethnicity, education level, marital status, smoking behavior, diabetes, hypertension, and cholesterol levels. Continuous covariates in our analysis included age, BMI, waist circumference, serum glucose, high density lipoprotein (HDL)-cholesterol, low density lipoprotein (LDL)-cholesterol, aspartate aminotransferase (AST), alanine aminotransferase (ALT), γ-glutamyl transpeptidase (GGT), serum albumin, serum creatinine, and uric acid. Detailed data on dietary fiber intake, CAP, and other variables are publicly accessible at http://www.cdc.gov/nchs/nhanes/.

### Statistical analysis

We utilized a weighted variance estimate approach to account for significant volatility in our data set. For categorical data, the weighted χ2 test was used to evaluate the differences between groups, and for continuous variables, the weighted linear regression model was employed. Utilizing a weighted multivariate regression model, we investigated the relationship between CAP and dietary fiber consumption. It’s worth noting that due to collinearity between serum glucose and diabetes status, as well as between HDL-cholesterol and LDL-cholesterol with high cholesterol levels, the multivariable regression equation did not include HDL-cholesterol, LDL-cholesterol, and serum glucose. Stratified multivariate regression analysis was used to carry out subgroup analysis. In order to examine the nonlinear association between dietary fiber intake and CAP, generalized additive models and smooth curve fits were employed. Upon discovering nonlinearity, we calculated the inflection point in the dietary fiber intake and CAP relationship using a recursive technique, followed by applying a two-piecewise linear regression model to both sides of this point. R (http://www.Rproject.org) and EmpowerStats (http://www.empowerstats.com) were used for all analyses, with a *P* value < 0.05 being regarded as statistically significant.

## Results

Our study included 5395 participants. The clinical features of CAP participants are shown in Table 1, which is arranged in columns for stratification. The group with severe steatosis is older, primarily male, married, and has a higher percentage of non-Hispanic White people. It also has a higher prevalence of cardiometabolic risk factors, including high blood pressure, smoking, diabetes, raised BMI, and hypercholesterolemia, than the non-NAFLD group. Increases in serum glucose, HDL-cholesterol and LDL-cholesterol, liver enzymes (AST, ALT, GGT), uric acid, and dietary fiber intake correlate with higher CAP values, while albumin levels decrease. There were no significant differences in the income to poverty ratio among the groups.

**Table 1:** Weighted characteristics of the study population based on controlled attenuated parameter (CAP)

	Non-NAFLD (CAP < 274, *n* = 3553)	NAFLD (274 ≤ CAP < 302, *n* = 857)	Severe steatosis (CAP ≥ 302, *n* = 1525)	*P* value
Age (years)	39.5 ± 22.2	50.0 ± 20.0	50.8 ± 18.0	< 0.001
Gender (%)				< 0.001
Men	1606 (45.2%)	388 (45.3%)	837 (54.9%)	
Women	1947 (54.8%)	469 (54.7%)	688 (45.1%)	
Race/Ethnicity (%)				< 0.001
Mexican American	390 (11.0%)	135 (15.8%)	265 (17.4%)	
Other Hispanic	343 (9.7%)	92 (10.7%)	156 (10.2%)	
Non-Hispanic White	1213 (34.1%)	298 (34.8%)	586 (38.4%)	
Non-Hispanic Black	1034 (29.1%)	222 (25.9%)	304 (19.9%)	
Other Race	573 (16.1%)	110 (12.8%)	214 (14.0%)	
Education level (%)				0.019
Less than high school	423 (17.2%)	157(20.7%)	261 (18.8%)	
High school	586 (23.9%)	181 (23.9%)	359 (25.9%)	
More than high school	1448(58.9%)	419(55.4%)	768(55.3%)	
Marital status (%)				< 0.001
Married/Living with Partner	1366 (55.6%)	449 (59.3%)	899 (64.6%)	
Widowed/Divorced/Separated	572 (23.3%)	190 (25.1%)	300 (21.6%)	
Never married	518 (21.1%)	118 (15.6%)	192 (13.8%)	
Income to poverty ratio	2.5 ± 1.7	2.6 ± 1.6	2.6 ± 1.6	0.231
BMI (kg/m^2^)	26.2 ± 6.1	31.7 ± 6.7	35.0 ± 7.8	< 0.001
Waist circumference (cm)	96.8 ± 17.4	99.8 ± 19.4	104.3 ± 19.1	< 0.001
Smoked at least 100 cigarettes in life				< 0.001
Yes	900 (33.7%)	268 (34.5%)	598 (41.8%)	
No	1773 (66.3%)	509 (65.5%)	833 (58.2%)	
Diabetes				< 0.001
Yes	242 (6.8%)	124 (14.5%)	378 (24.8%)	
No	3246 (91.4%)	709 (82.8%)	1086 (71.2%)	
Borderline	64 (1.8%)	23 (2.7%)	61 (4.0%)	
Hypertension				< 0.001
Yes	800 (26.9%)	339 (42.0%)	708 (48.4%)	
No	2171 (73.1%)	468 (58.0%)	756 (51.6%)	
High cholesterol level				< 0.001
Yes	786 (26.5%)	314 (39.1%)	632 (43.5%)	
No	2176 (73.5%)	489 (60.9%)	820 (56.5%)	
Serum glucose (mmol/L)	5.8 ± 1.5	6.2 ± 1.9	7.2 ± 2.7	< 0.001
HDL-Cholesterol (mmol/L)	1.4 ± 0.4	1.3 ± 0.4	1.2 ± 0.3	< 0.001
LDL-Cholesterol (mmol/L)	2.6 ± 0.9	2.9 ± 0.9	2.8 ± 0.9	< 0.001
AST (IU/L)	20.0 ± 9.3	21.3 ± 12.8	23.0 ± 13.8	< 0.001
ALT (IU/L)	17.2 ± 10.8	21.9 ± 14.6	27.8 ± 19.7	< 0.001
GGT (IU/L)	21.8 ± 25.4	31.4 ± 47.3	36.6 ± 39.5	< 0.001
Serum albumin (g/L)	41.4 ± 3.4	40.5 ± 3.2	40.3 ± 3.4	< 0.001
Serum creatinine (mg/dl)	0.8 ± 0.4	0.9 ± 0.3	0.9 ± 0.4	< 0.001
Uric acid (mg/dl)	5.0 ± 1.3	5.5 ± 1.4	5.8 ± 1.5	< 0.001
Dietary fiber intake (g/day)	14.4 ± 8.8	14.6 ± 8.3	15.0 ± 8.7	0.005

Mean ± SD for continuous variables: the *P* value was calculated by the weighted linear regression model. (%) for categorical variables: the *P* value was calculated by the weighted chi-square test

Table 2 shows the results of the multivariate regression analysis. In the unadjusted Model 1, daily fiber intake showed no significant association with CAP (β = 0.00, 95% CI: -0.19, 0.19, *P* = 0.9900). But when age, sex, and race/ethnicity were taken into account, Model 2 showed a significant correlation between increased fiber consumption and decreased CAP (β=-0.43, 95% CI: -0.61, -0.25, *P* < 0.0001). Even after accounting for extra factors, Model 3’s negative connection persisted (β=-0.19, 95% CI: -0.38, -0.01, *P* = 0.0398). Analysis by quartiles of dietary fiber intake in Model 3 showed that the highest quartile (Q4) had significantly lower CAP than the lowest quartile (Q1) (β = -5.78, 95% CI: -10.40, -1.15, *P* = 0.0144), demonstrating a significant linear trend (P for trend = 0.026). The inverse link between dietary fiber consumption and CAP remained significant for women (β=-0.29, 95% CI: -0.56, -0.02, *P* = 0.0329) and Whites (β=-0.39, 95% CI: -0.69, -0.09, *P* = 0.0113) in Model 3, according to subgroup analyses by age, sex, and race/ethnicity in Table 2.

**Table 2:** The association between dietary fiber intake (g/day) and controlled attenuation parameter (dB/m)

	Model 1 β (95% CI) *P* value	Model 2 β (95% CI) *P* value	Model 3 β (95% CI) *P* value
Dietary fiber intake (g/day)	0.00 (-0.19, 0.19) 0.9900	-0.43 (-0.61, -0.25) < 0.0001	-0.19 (-0.38, -0.01) 0.0398
Dietary fiber intake quartile			
Q1 (0.20–8.40 g/day)	Reference	Reference	Reference
Q2 (8.41–12.80 g/day)	1.53 (-3.18, 6.25) 0.5237	-1.19 (-5.67, 3.29) 0.6018	0.12 (-4.49, 4.73) 0.9590
Q3 (12.81–18.75 g/day)	6.28 (1.67, 10.89) 0.0076	0.17 (-4.23, 4.57) 0.9391	1.63 (-2.84, 6.11) 0.4748
Q4 (18.76–72.30 g/day)	0.56 (-4.10, 5.23) 0.8125	-10.25 (-14.76, -5.73) < 0.0001	-5.78 (-10.40, -1.15) 0.0144
P for trend	0. 410	< 0.001	0.026
Subgroup analysis stratified by age			
< 45 years	-0.19 (-0.47, 0.08) 0.1604	-0.39 (-0.66, -0.12) 0.0049	-0.16 (-0.45, 0.13) 0.2774
≥ 45 and < 60 years	0.06 (-0.31, 0.44) 0.7405	-0.17 (-0.55, 0.22) 0.3901	-0.07 (-0.42, 0.27) 0.6779
≥ 60 years	-0.26 (-0.60, 0.08) 0.1301	-0.44 (-0.78, -0.10) 0.0116	-0.26 (-0.60, 0.07) 0.1244
Subgroup analysis stratified by sex			
Men	0.11 (-0.15, 0.37) 0.3958	-0.33 (-0.58, -0.07) 0.0113	-0.10 (-0.35, 0.15) 0.4256
Women	-0.33 (-0.60, -0.05) 0.0190	-0.55 (-0.81, -0.29) < 0.0001	-0.29 (-0.56, -0.02) 0.0329
Subgroup analysis stratified by race/ethnicity			
Mexican American	0.56 (0.10, 1.02) 0.0173	0.10 (-0.36, 0.55) 0.6770	0.26 (-0.18, 0.70) 0.2429
Other Hispanic	0.42 (-0.16, 1.01) 0.1583	0.26 (-0.31, 0.84) 0.3662	-0.01 (-0.58, 0.56) 0.9799
Non-Hispanic White	-0.32 (-0.65, 0.01) 0.0594	-0.72 (-1.03, -0.40) < 0.0001	-0.39 (-0.69, -0.09) 0.0113
Non-Hispanic Black	0.02 (-0.39, 0.43) 0.9059	-0.26 (-0.66, 0.14) 0.2005	0.11 (-0.35, 0.57) 0.6323
Other Race	0.19 (-0.20, 0.58) 0.3468	-0.19 (-0.56, 0.19) 0.3305	0.14 (-0.32, 0.60) 0.5449

Model 1: no covariates were adjusted

Model 2: age, sex, and race/ethnicity were adjusted

Model 3: age, sex, race/ethnicity, education level, marital status, body mass index, Waist circumference, smoking behavior, and the existence of diabetes, hypertension, and high cholesterol level, aspartate aminotransferase, alanine aminotransferase, γ- glutamyl transpeptidase, serum albumin, serum creatinine and uric acid were adjusted

In the subgroup analysis stratified by age, sex and race/ethnicity, the model is not adjusted for age, sex and race/ethnicity, respectively

**Fig. 2:**
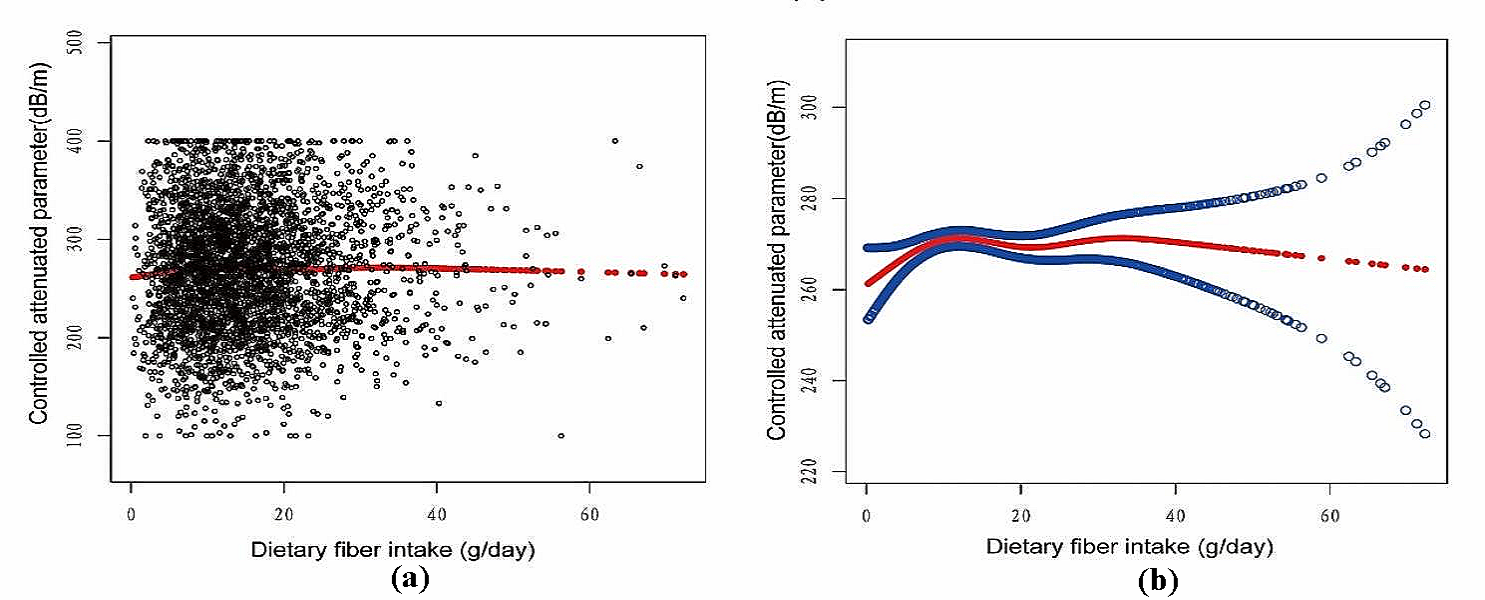
The association between Dietary fiber intake and controlled attenuation parameter. (**a**) Each black point represents a sample. (**b**) Solid rad line represents the smooth curve fit between variables. Blue bands represent the 95% of confidence interval from the fit. Age, sex, race/ethnicity, education level, marital status, body mass index, Waist circumference, smoking behavior, and the existence of diabetes, hypertension, and high cholesterol level, aspartate aminotransferase, alanine aminotransferase, γ- glutamyl transpeptidase, serum albumin, serum creatinine and uric acid were adjusted

**Fig. 3:**
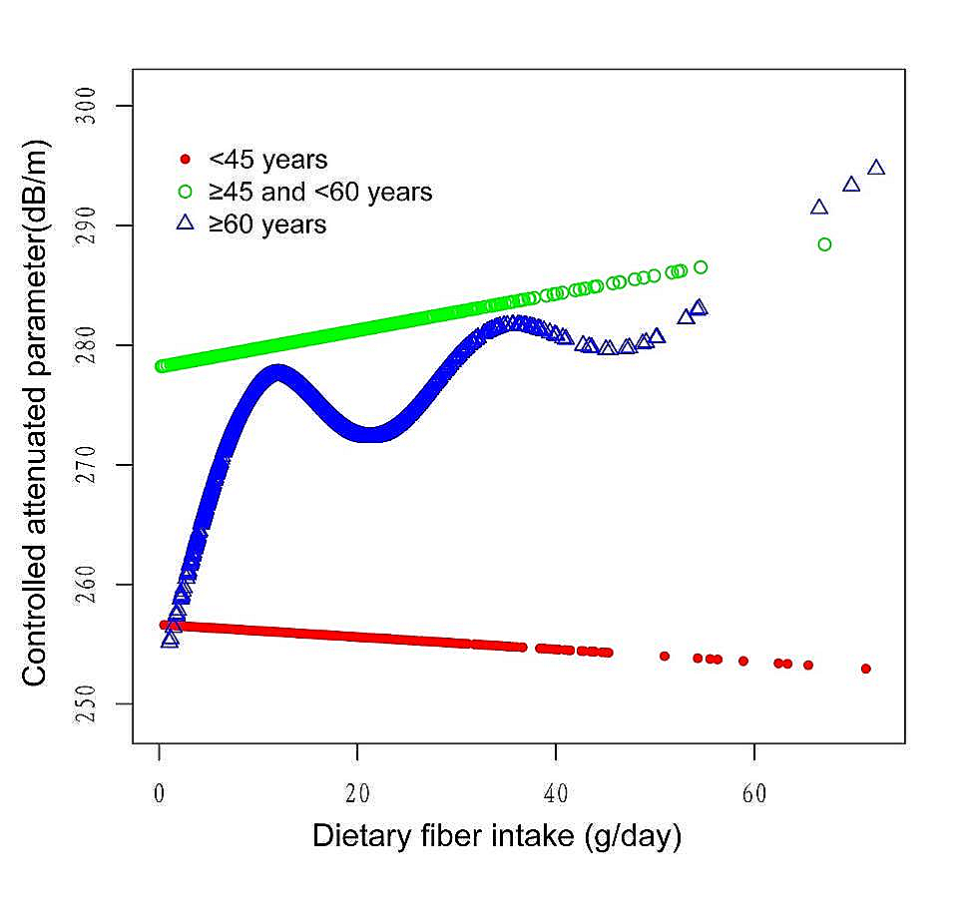
The association between Dietary fiber intake and controlled attenuation parameter stratified by age. Sex, race/ethnicity, education level, marital status, body mass index, Waist circumference, smoking behavior, and the existence of diabetes, hypertension, and high cholesterol level, aspartate aminotransferase, alanine aminotransferase, γ- glutamyl transpeptidase, serum albumin, serum creatinine and uric acid were adjusted

**Fig. 4:**
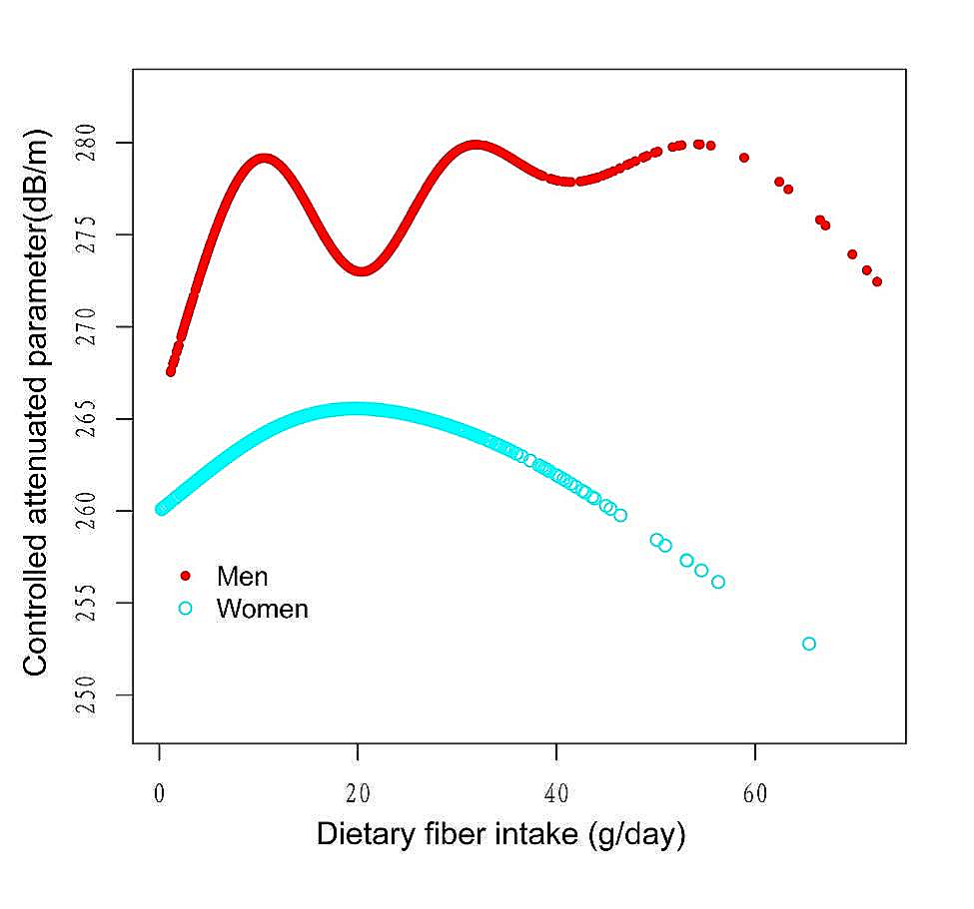
The association between Dietary fiber intake and controlled attenuation parameter stratified by sex. Age, race/ethnicity, education level, marital status, body mass index, Waist circumference, smoking behavior, and the existence of diabetes, hypertension, and high cholesterol level, aspartate aminotransferase, alanine aminotransferase, γ- glutamyl transpeptidase, serum albumin, serum creatinine and uric acid were adjusted

**Fig. 5:**
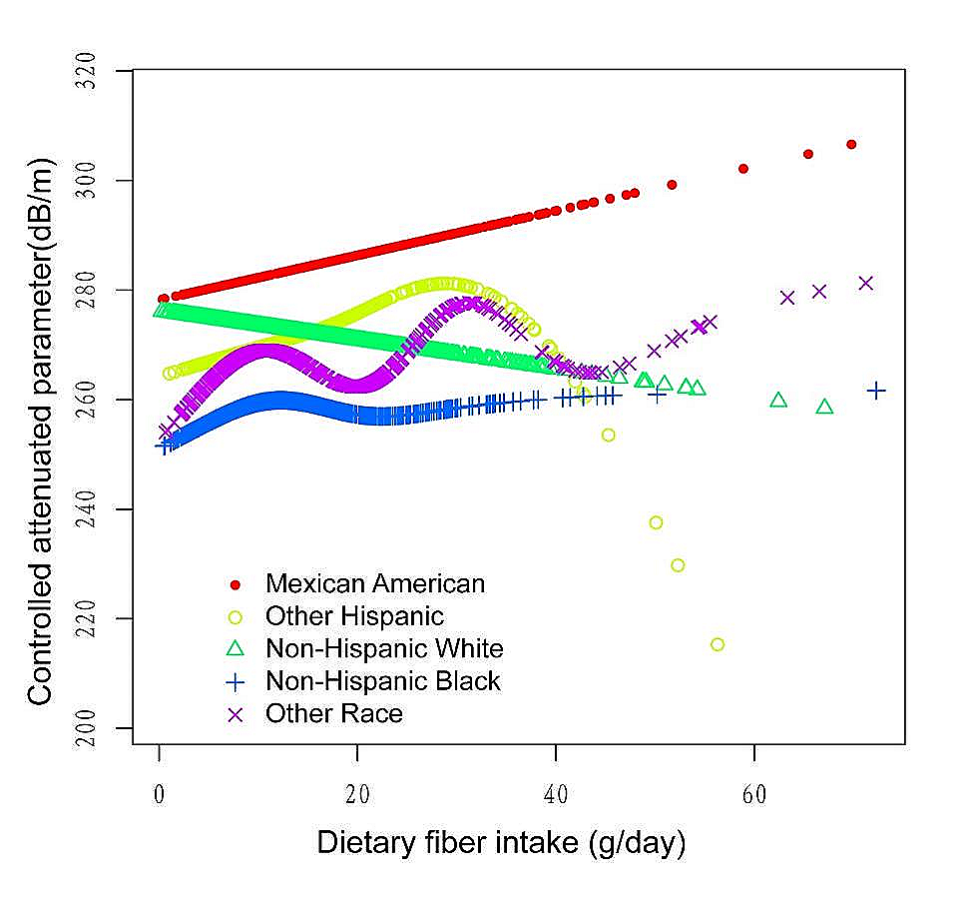
The association between Dietary fiber intake and controlled attenuation parameter stratified by race/ethnicity. Age, sex, education level, marital status, body mass index, Waist circumference, smoking behavior, and the existence of diabetes, hypertension, and high cholesterol level, aspartate aminotransferase, alanine aminotransferase, γ- glutamyl transpeptidase, serum albumin, serum creatinine and uric acid were adjusted

Figures 2, 3, 4 and 5 illustrate smooth curve fits and generalized additive models, demonstrating an inverted U-shaped relationship between dietary fiber intake and CAP in women and other race. In women, the two-piecewise linear model indicated a dietary fiber intake inflection point at 13.80 g/day (Table 3). Below this threshold, there was no significant association between dietary fiber intake and CAP levels (β = 0.44, 95% CI: -0.26, 1.13, *P* = 0.2172). Above 13.80 g/day, a higher dietary fiber intake significantly correlated with lower CAP levels (β=-0.62, 95% CI: -1.01, -0.23, *P* = 0.0021). The statistically significant likelihood ratio test between the standard linear and two-piecewise linear models supports a threshold effect (*P* = 0.025). Similarly, in other race, inflection points occur at a dietary fiber intake of 33.45 g/day, as evidenced by a significant likelihood ratio (*P* = 0.004) (Table 4). For dietary fiber intakes below 33.45 g/day, each 1 g/day increase was related to a 0.65 dB/m raise in CAP (95% CI: -0.08, 1.39, *P* = 0.0801); by comparison, for individuals with a dietary fiber intake > 33.45 g/day, a 1 g/day upregulation was connected with a 3.46 dB/m drop in CAP (95%CI: -5.95, -0.97, *P* = 0.0069).

**Table 3:** Threshold effect analysis of dietary fiber intake on controlled attenuation parameter in women using the two-piecewise linear regression model

controlled attenuation parameter	Adjusted β (95% CI), *P* value
Women	
Fitting by the standard linear model	-0.29 (-0.56, -0.02) 0.0329
Fitting by the two-piecewise linear model	
Inflection point	13.80
Dietary fiber < 13.80 (g/day)	0.44 (-0.26, 1.13) 0.2172
Dietary fiber > 13.80 (g/day)	-0.62 (-1.01, -0.23) 0.0021
Log likelihood ratio	0.0250

Age, race/ethnicity, education level, marital status, body mass index, Waist circumference, smoking behavior, and the existence of diabetes, hypertension, and high cholesterol level, aspartate aminotransferase, alanine aminotransferase, γ- glutamyl transpeptidase, serum albumin, serum creatinine and uric acid were adjusted

**Table 4:** Threshold effect analysis of dietary fiber intake on controlled attenuation parameter in other Race using the two-piecewise linear regression model

controlled attenuation parameter	Adjusted β (95% CI), *P* value
Other Race	
Fitting by the standard linear model	-0.01 (-0.58, 0.56) 0.9799
Fitting by the two-piecewise linear model	
Inflection point	33.45
Dietary fiber < 33.45 (g/day)	0.65 (-0.08, 1.39) 0.0801
Dietary fiber > 33.45 (g/day)	-3.46 (-5.95, -0.97) 0.0069
Log likelihood ratio	0.004

Age, sex, education level, marital status, body mass index, Waist circumference, smoking behavior, and the existence of diabetes, hypertension, and high cholesterol level, aspartate aminotransferase, alanine aminotransferase, γ- glutamyl transpeptidase, serum albumin, serum creatinine and uric acid were adjusted

## Discussion

This study analyzed data from the NHANES in the United States from 2017 to 2020. Using the CAP for direct assessment, the study explored the relationship between dietary fiber intake and hepatic steatosis. Multivariate logistic regression analysis, adjusting for potential confounding factors, revealed that higher dietary fiber intake is associated with lower CAP values. This suggests that dietary fiber consumption and hepatic steatosis are negatively correlated, especially in women and White individuals.

Our findings are consistent with research from different populations across various countries and animal models. For instance, a study conducted in the Netherlands showed that higher liver fat indices were associated with lower dietary fiber intake [24]. Likewise, an Iranian case-control study found that NAFLD patients consumed less dietary fiber than healthy individuals [23]. NAFLD and total dietary fiber consumption were also found to be negatively correlated in another Chinese investigation [25]. Animal experiments further support these observations. One study observed that high-viscosity dietary fiber intake could reduce obesity and liver fat changes caused by a high-fat diet [36]. Another experiment, simulating a Western diet in mice, showing that a long-term high-fat, low-fiber diet (HFFD) resulted in liver fat changes [37]. Collectively, these studies suggest a protective effect of dietary fiber against liver fat changes, underscoring its potential significance in dietary guidelines for liver health.

Nevertheless, an Israeli cross-sectional study revealed no notable disparities in dietary fiber consumption between NAFLD patients and healthy individuals [26]. This outcome might be attributed to the study’s lack of adjustment for confounding factors. Similarly, a case-control study involving 36 Chinese individuals indicated no variance in dietary fiber intake between those with NAFLD and healthy subjects [27]. The limited sample size and incomplete adjustment for confounding factors could explain this observation. Additionally, a recent cross-sectional study of 3,882 elderly participants did not find a consistent link between dietary fiber consumption and NAFLD [28]. The limitations present in these studies contribute to the inconclusive evidence about the link between dietary fiber intake and alterations in hepatic steatosis.

Insufficient dietary fiber is linked to an increased risk of hepatic steatosis, as it plays a crucial role in regulating lipid synthesis pathways and preventing liver fat alterations [38]. Dietary fiber could also mitigate hepatic steatosis via the liver’s G-protein-coupled receptor (GPR) 41/43-calmodulin-dependent protein kinase II (CaMKII), histone deacetylase 1 (HDAC1)-cyclic adenosine monophosphate response element-binding protein (CREB) pathway [37]. Furthermore, studies suggest that dietary fiber intake is associated with regulating glucose homeostasis and gut microbiota, which may help prevent liver fat changes [39–41].

Furthermore, in smooth curve fitting and threshold effect analysis across different ages, genders, and races, we observed a nonlinear relationship between dietary fiber intake and CAP in women and other race, with inflection points at 13.8 g/day and 33.45 g/day respectively. To our knowledge, this might be the first study to uncover an inverse U-shaped relationship between dietary fiber intake and hepatic steatosis in American women and other race American. This indicates that these groups need to exceed certain fiber intake thresholds for dietary fiber to benefit liver health. Clinically, this suggests the advantage of personalized dietary recommendations, particularly for women and other racial groups, emphasizing increased intake of high-fiber foods like fruits and vegetables to reduce the risk of liver fat accumulation.

The relationship between dietary fiber and hepatic steatosis is intricate, potentially involving various mechanisms such as insulin resistance, hepatic lipid metabolism, and changes in gut microbiota [42]. According to the research [43], the consumption of total fiber, insoluble fiber, and soluble fiber differs among different ethnicities and genders, leading to varying risks of insulin resistance and hypertension. Additionally, dietary fiber intake may play a role in modulating gut microbiota activity, which could have different effects on hepatic steatosis depending on gender [44]. Variations in risk factors between ethnicities can also be explained by differences in genetic predisposition, obesity rates, and other factors. However, further prospective studies with substantial sample sizes are needed to elucidate the complex relationship between dietary fiber and hepatic steatosis among different genders and ethnicities. In our research, the scale of the cohort strengthens the findings, as NHANES aims to generate nationally representative estimates.

Our study boasts numerous strengths. Firstly, we used a large and nationally representative sample from the United States, minimizing selection bias. Secondly, we directly measured liver fat content using the reliable CAP method, rather than indirect assessment through imaging or biochemical markers, enhancing the accuracy of our findings. Furthermore, our comprehensive analysis methods, including subgroup analysis for women and Whites, confirm the overall results, increasing the reliability of our conclusions. Most notably, employing curve fitting and nonlinear models to explore the non-linear relationship between dietary fiber and liver fat changes highlighted threshold effects.

Nonetheless, there are limitations to our study. First off, as a cross-sectional study, it cannot establish causality. Additionally, the dietary fiber intake data, derived from two 24-hour dietary recalls, may be potentially susceptible to reporting bias. Finally, the fact that participants were exclusively from the United States limits the generalizability of the results to other populations.

## Conclusions

Our research indicates that in the majority of Americans, there is an inverse relationship between dietary fiber intake and changes in hepatic steatosis. In females and individuals of other races, this relationship demonstrates an inverted U-shaped curve, indicative of a threshold effect. This implies that a specific amount of dietary fiber is required for beneficial effects on liver health in these groups. The findings of this study hold potential significance for clinical nutrition interventions, personalized dietary guidance, and advancing research into the diet-disease mechanism relationship.

## Data Availability

All NHANES data for this study are publicly available and can be found here: https://wwwn.cdc.gov/nchs/nhanes.
